# Imaging the Predicted Isomerism of Oligo(aniline)s: A Scanning Tunneling Microscopy Study

**DOI:** 10.1002/smll.201500511

**Published:** 2015-03-18

**Authors:** James O Thomas, Hugo D Andrade, Benjamin M Mills, Neil A Fox, Heinrich J K Hoerber, Charl F J Faul

**Affiliations:** Bristol Centre for Functional Nanomaterials, Centre for NSQI, University of BristolTyndall Avenue, BS8 1FD, UK; H. H. Wills Physics Laboratory, University of BristolTyndall Avenue, BS8 1TL, UK E-mail: h.hoerber@bristol.ac.uk; School of Chemistry, University of BristolCantock's Close, BS8 1TS, UK E-mail: charl.faul@bristol.ac.uk

**Keywords:** density functional calculations, isomers, oligomers, scanning tunneling microscopy, thin films

Understanding the organization of π-conjugated functional organic materials in thin films is of crucial importance to fully exploit their tuneable properties for applications such as field-effect transistors[1] and photovoltaics.[2] The optoelectronic properties of such thin films depend on both the molecular architecture as well as the supramolecular structure (including aspects such as π-overlap and packing geometries) of the films.[3,4] Furthermore, the self-assembly and properties of multilayered thin films crucially depend on the interaction of the first layer with the underlying substrate.[5] Unless the material exhibits some degree of order, spatially averaged techniques, e.g., grazing-incidence X-ray scattering (GIXS) and low-energy electron diffraction (LEED), cannot offer any information on the structure of this layer, other than the lack of periodicity. Scanning tunneling microscopy (STM), however, provides real-space imaging at the molecular level,[6] and can offer direct insight into the relationship between molecular and local architecture, the ability to organize on a supramolecular level, and the influence of the substrate on such organization, or lack thereof.[7]

Since its discovery, poly(aniline) (PANI) has been widely investigated owing to its rich redox behavior and three distinct oxidation states, the leucoemeraldine base (LEB), emeraldine base (EB), and pernigraniline base (PB) states. PANI also possesses a conducting emeraldine salt (ES), easily accessible by acid doping (from EB) or oxidative doping (from LEB). Thin films of PANI have been investigated for use in flash-memory[8] and sensors[9] and have found commercial application in printed circuit board manufacturing and anti-static coatings. However, conductivity in PANI has been observed at values significantly below the theoretically predicted maximum value,[10] due to molecular and mesoscopic structural defects.[11] Values as high as 10^3^ S cm^−1^ have only been recorded in exceptional cases. As model systems for PANI, tetra(aniline) (TANI) and its derivatives can be prepared as chemically pure, discrete units that are soluble in a range of organic solvents and amenable to a host of preparation methods, whilst retaining the unique redox properties of PANI.[12] Despite the short conjugated chain length, microstructures of ES TANI, prepared by acid-doping of EB structures, are able to approach the conductivities of PANI, owing to high sample crystallinity.[13]

In this report, monolayer formation of EB TANIs on a Cu(110) surface is investigated at the single-molecule level using STM. MacDiarmid et al. predicted, due to the presence of the quinoid ring in the molecular backbone, a number of different positional, geometric, and conformational isomers for EB TANIs (**Figure** **1**).[14] Whereas the positional isomers have been confirmed and observed with NMR in solution,[15] and related to reports of inconsistent conductivity in PANI, the *cis*/*trans* isomers have not been directly experimentally observed in TANIs. The *cis*/*trans* isomerization produces a much more profound 2D change in molecular shape, and is likely to significantly reduce the ability of these materials to form crystalline structures. Here we present STM data showing, for the first time, the presence of these *cis*/*trans* isomers in two different TANI-based materials, and relate their presence to the lack of structure formation and consequent absence of structural data in the literature.

**Figure 1:**
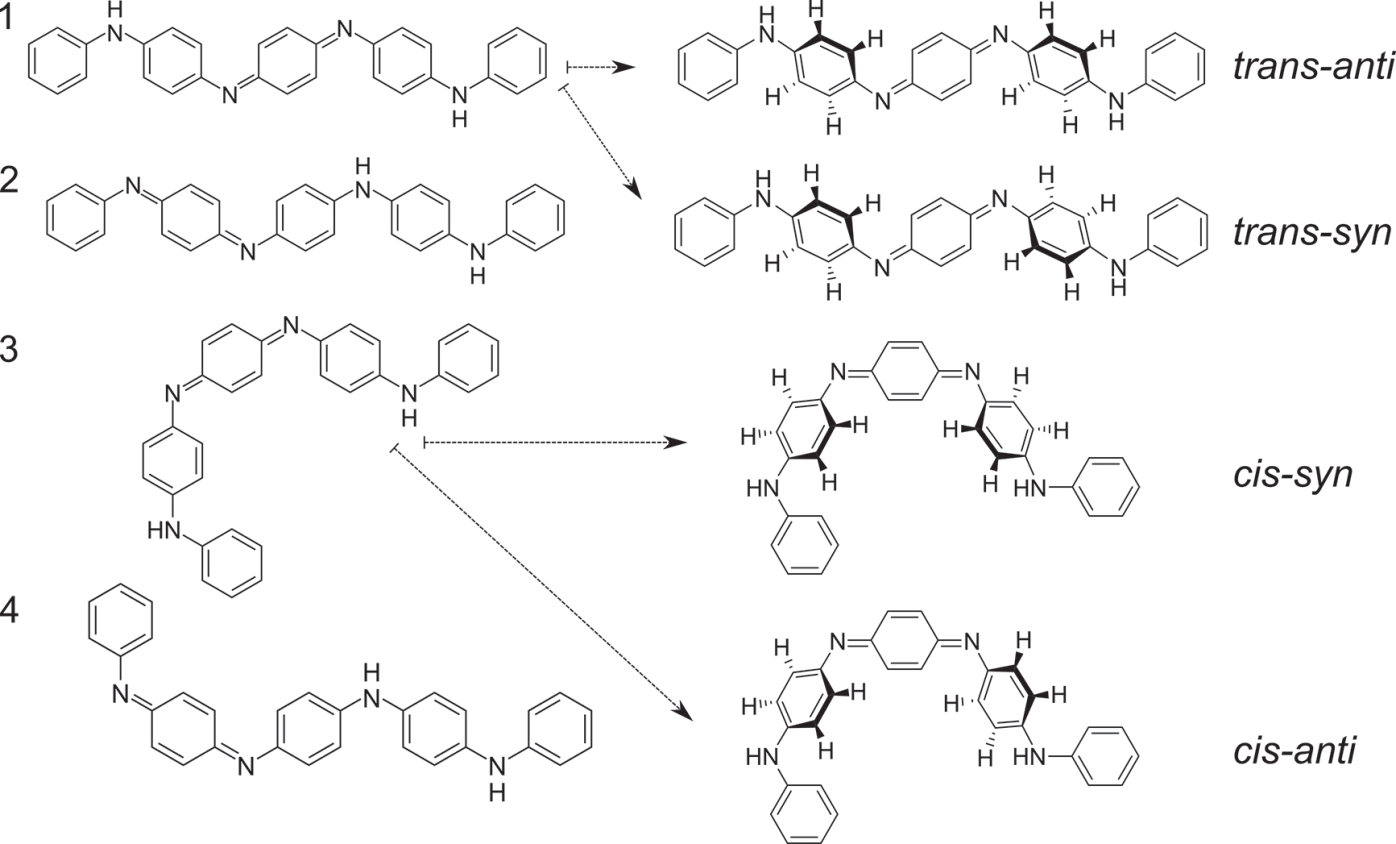
Four possible isomers of Ph/Ph TANI, with each of the two positional *trans* isomers (1 and 2) having a corresponding *cis* isomer (3 and 4). *Syn*/*anti* conformers for 1 and 3 are shown explicitly and analogues for 2 and 4 also exist (see the Supporting Information).

Monolayers of phenyl capped TANI (Ph/Ph TANI) were formed by evaporation onto a clean Cu(110) surface in ultra-high vacuum (UHV) conditions (see the Supporting Information for experimental details). Diffusion of the molecules rendered imaging impossible at room temperature; cooling the surface down to 30 K using a continuous-flow liquid helium cryostat however ensured immobilization and enabled imaging of the Ph/Ph TANI. **Figure** **2**a shows a Ph/Ph TANI film at 30 K, with a clear lack of long-range order. Higher resolution images in Figure 2b,c show the presence of *cis*/*trans* isomers within this disordered layer, with gas-phase DFT-calculated optimized structures shown alongside in Figure 2d,e (additional calculated structures can be found in the Supporting Information). The calculated and optimized structures and STM images show similar lengths: both the *trans* isomer (**1** or **2**, indistinguishable at this resolution) shown in Figure 2b and the optimized *trans* structure shown in Figure 2d have lengths of 2.3 nm. The length of the *cis* isomer in Figure 2c (isomer **3**) also corresponds well, with a length of 2.2 nm and an angle of α_STM_ ≈ 43° slightly reduced from that observed in the optimized structure in Figure 2e (α_DFT_ ≈ 48°, *length*_DFT_ of 2.3 nm).[16] The structures shown in Figure 2b,c can be assigned unambiguously as *cis* and *trans* isomers of Ph/Ph TANI, and highlights the inability of these *cis*/*trans* EB TANIs to self-assemble. It is noteworthy that isomer **4**, with a lower energy barrier and “intermediate” conformation, could not be identified unambiguously within the monolayer.

**Figure 2:**
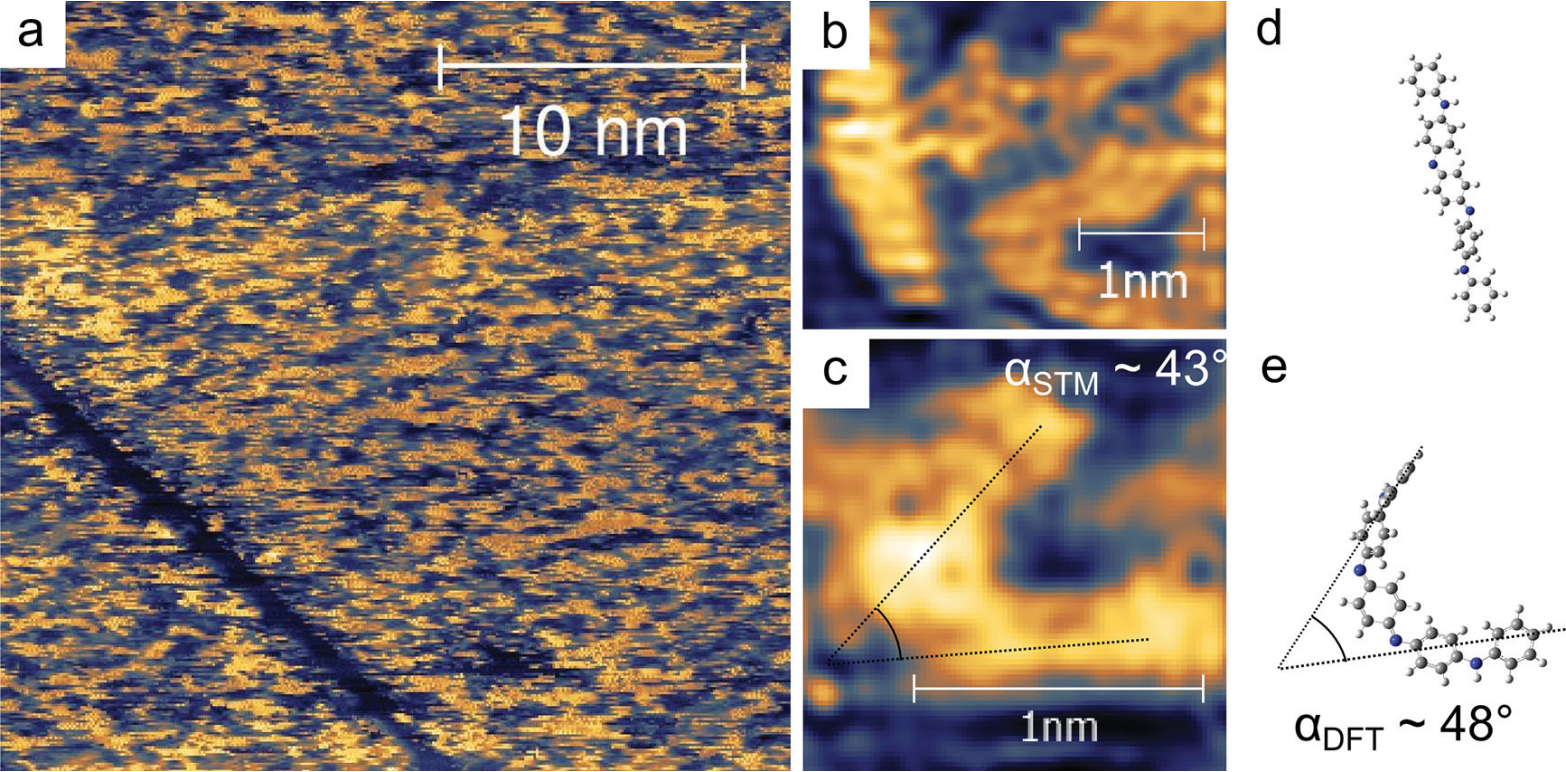
a) STM image of a disordered layer of Ph/Ph TANI on Cu(110). b,c) *Trans* and *cis* isomers present within the layer. d,e) Calculated optimized gas-phase structures of *trans* and *cis-*Ph/Ph TANI. All images were taken with the sample at 30 K. High frequency noise was removed by applying Fourier filtering to images b) and c); this did not affect the molecular shape or size. Scanning conditions: a): *I* = 47 pA, *V* = 0.75 V; b): *I* = 25 pA, *V* = 0.65 V; c): *I* = 20 pA, *V* = 1 V.

From earlier studies, it was expected that the copper substrate would play a role in stabilizing the structures formed, with the electron-rich metal likely to form a strong interaction with the electron-deficient quinoid ring. Experimental observation corroborated such behavior: whilst annealing the EB Ph/Ph TANI monolayers above 370 K in an attempt to traverse energy barriers and access a thermodynamically more stable state, desorption of the majority of the material, possibly via fragmentation, occurred, leaving isolated double-lobed structures on the surface. These double-lobed structures were observed at both 30 K (**Figure** **3**a) and 300 K (Figure 3b). The double-lobed structures at 300 K could be imaged for over 30 min without appreciable diffusion, suggesting some degree of chemisorption had taken place. It is proposed that the two lobes are the –C_6_H_4_–NH–C_6_H_5_ fragments (“arms”) of TANI, shown in Figure 3d, with the quinoid ring planar to the surface (similar to behavior calculated for azobenzene molecules on copper).[17] The line profiles over the structures also support this conclusion, with the apparent size reduced from ≈2.3 to ≈2.0 nm, suggesting that the molecule is anchored and able to curl into the *z*-direction.

**Figure 3:**
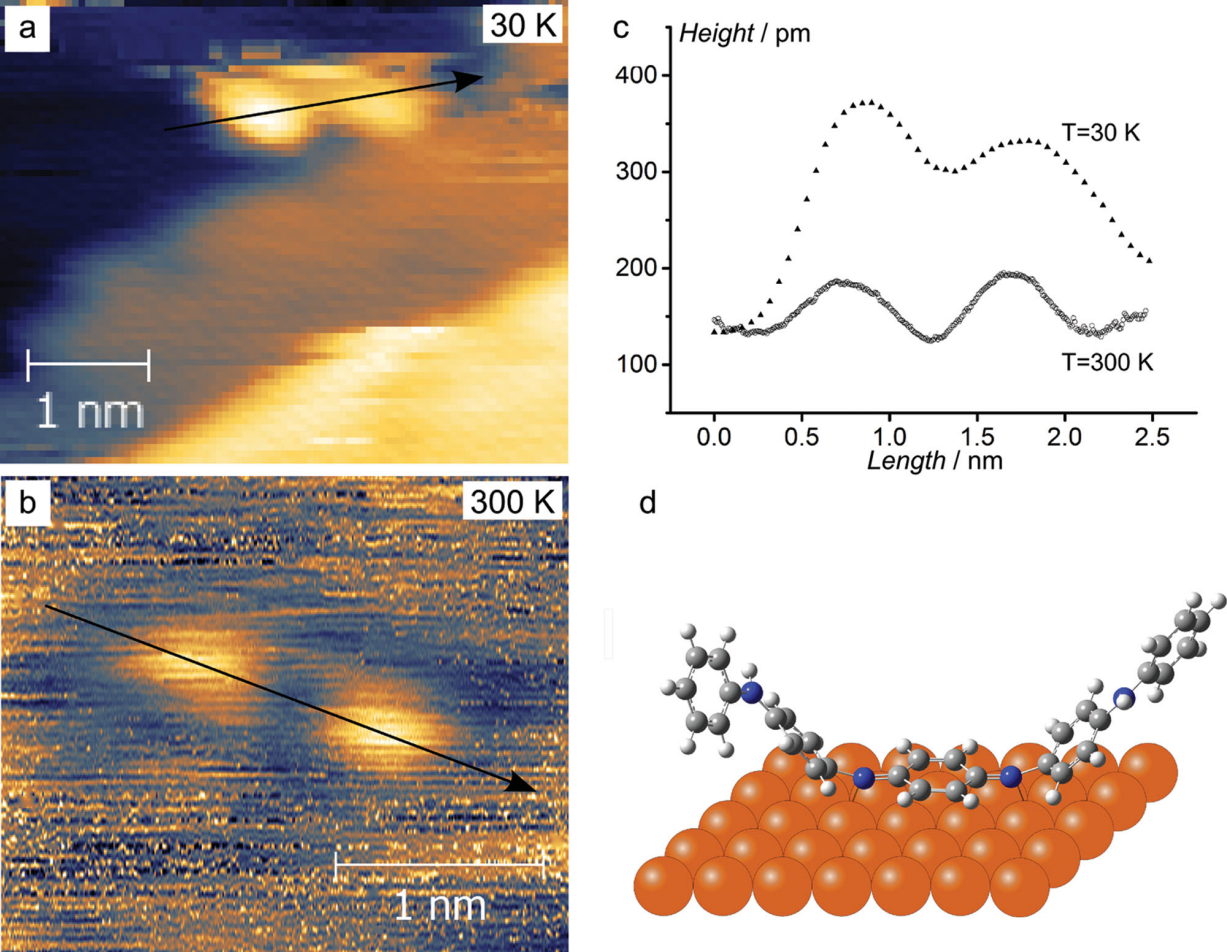
a,b) show individual double-lobed Ph/Ph TANI at 30 K and 300 K, respectively. Line profiles, indicated by the arrows in a) and b), are plotted in c). d) The profiles show a decrease in molecular size with respect to preannealed samples, indicating that the molecules were no longer lying flat, but with some degree of chemisorption, as postulated in the schematic. Scanning conditions: a) *I* = 100 pA, *V* = 0.75 V; b) *I* = 50 pA, *V* = −1 V.

The growth of oligomeric, as well as polymeric materials, into 3D structures is strongly directed by the initial 2D self-assembly of molecules on the substrate. This initial interaction can dominate the final structure, and consequently the properties, of such films.[5a,^18^] Confinement of *bis*(2-ethylhexyl) hydrogen phosphate (BEHP)-doped ES Ph/Ph TANI to thin films and analysis by GIXS has shown ES state films form a lamellar structure with long range order of up to 50 molecular layers with a d-spacing of 2.15 nm; however, the analysis of EB Ph/Ph TANI thin films prepared in the same manner shows minimal evidence of ordering.[19] Crystal structures of HClO_4_-doped and HBF_4_-doped ES Ph/Ph TANI,[20] as well as LEB Ph/Ph TANI,[21] have been reported; whilst ES and LEB TANI can exhibit conformational isomerism due to out-of-plane twisting of the benzenoid rings, crucially, it seems, the positional and geometric isomers are no longer present. After inspection of the STM data presented here, it is hardly surprising that no EB Ph/Ph TANI has been crystallized and that structural studies on thin films show little order. We believe our experimental results provide hints toward an explanation of the unfulfilled properties of PANI and TANI systems, as discussed by MacDiarmid.[14]

Previous work has shown that the addition of an alkyl tail moiety to TANI leads to the formation of crystalline microstructures in the EB state.[22] We therefore prepared a dodecyl-functionalized TANI, Ph/C_12_ TANI, which was deposited in the same fashion as the Ph/Ph TANI (**Figure** **4**). The addition of an alkyl chain, connected by an amide bond, was however not sufficient to induce ordered self-assembled monolayers, but did provide enough of an attraction and registration with the underlying Cu(110) substrate to allow imaging at room temperature and gentle thermal annealing (up to 400 K). Again it was evident that the molecules formed a disordered layer with a range of 2D shapes. The straighter, *trans* molecules showed limited alignment along the step edges. Examples of structures optimized by DFT are presented in Figure 4(see also the Supporting Information for a more comprehensive list), and presented alongside tentative matches to shapes found within the molecular monolayer. DFT-optimized structures of Ph/C_12_ TANI have a molecular length of 3.4 nm, which compares well with the length measured from STM images of 3.5 nm for both isomers given in Figure 4c, and 3.4 nm for the two molecules shown in Figure 4d. Internal angles of these isomers compared with the calculated values show some deviation from the experimental data, and are summarized in **Table** **1**. This establishes that the calculated molecular lengths and those measured using STM correspond excellently, as expected. However some angles measured are subject to significant variations, likely due to interactions with coplanar molecules and the underlying substrate, as was seen for Ph/Ph TANI. Another feature of the Ph/C_12_ TANI system that Figure 4c,d displays is the additional *cis*/*trans* isomerism around the amide bond. This feature is observed despite the lower thermodynamic stability of the *cis*-amide in gas phase structures (typically by around ≈15 kJ mol^−1^), and adds another array of potential 2D shapes. The nonequilibrium nature of thermal evaporation as a sample preparation technique is also highlighted by these observations.

**Table 1:** Measured and calculated lengths of Ph/Ph TANI and Ph/C_12_ TANI molecules displayed in Figures 2 and 4

Molecule	Length	α	β	γ	δ	ε	ζ
Ph/Ph TANIa)	2.3 nm	39	…	…	…	…	…
Ph/Ph TANIb)	2.3 nm	43	…	…	…	…	…
Ph/C_12_ TANIa)	3.5 nm	…	88	93	111	95	92
Ph/C_12_ TANIb)	3.4 nm	…	95	101	115	91	96

a) Measured from STM images;

b) Calculated using DFT. For values where >1 measurements were taken, the values are averaged.

**Figure 4:**
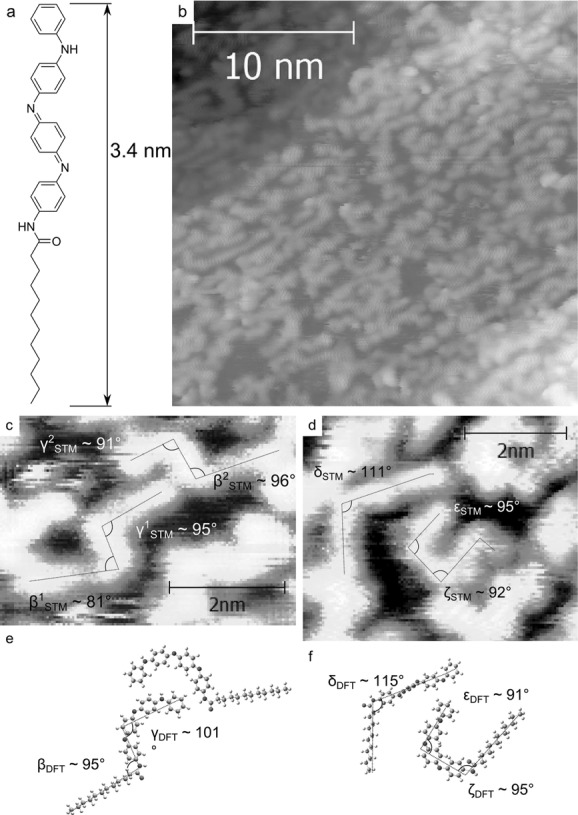
a) Ph/C_12_ TANI molecular structure. b) STM image at 300 K of a disordered monolayer containing a variety of isomers, including some evidence of fragmentation. Higher resolution scans are shown in c) and d), respectively. e,f) show optimized structures similar in shape to the STM data, and angles of these shapes are indicated on the STM data and optimized structures. Scanning conditions for b), c), and d): *I* = 100 pA, *V* = 0.35 V.

In conclusion, monolayers of EB state Ph/Ph and Ph/C_12_ TANI were deposited under UHV conditions and shown to form disordered structures. The lack of order in these monolayers can be attributed to number of isomers available to this particular oxidation state of TANIs.[14] These isomers were revealed for the first time by our low temperature UHV-STM investigations, and fully supported by calculations. These results highlight the importance of isomerism in the self-assembly of small molecules, and the influence of the presence of isomers on the formation of ordered 3D structures, i.e., aspects that should be considered during the design of small molecules for thin film applications. In addition, the data support predicted reasons for the inability of PANI to achieve its theoretical conductivity and therefore fulfil its potential, despite intensive research efforts.

## Supporting Information

As a service to our authors and readers, this journal provides supporting information supplied by the authors. Such materials are peer reviewed and may be re-organized for online delivery, but are not copy-edited or typeset. Technical support issues arising from supporting information (other than missing files) should be addressed to the authors.
