# Supplementary material for: Imaging the Predicted Isomerism of Oligo(aniline)s: A Scanning Tunneling Microscopy Study
Source: Small. 2015 Mar 18;11(28):3430–4. doi: 10.1002/smll.201500511 (PMC4692098; doi:10.1002/smll.201500511)
Supplement: Supplementary file 1 — Supplementary [file smll0011-3430-sd1.pdf]

Copyright WILEY-VCH Verlag GmbH & Co. KGaA, 69469 Weinheim, Germany, 2015.

NANO small MICRO

Supporting Information

for *Small*, DOI: 10.1002/smll.201500511

Imaging the Predicted Isomerism of Oligo(aniline)s: A Scanning Tunneling Microscopy Study

James O. Thomas, Hugo D. Andrade, Benjamin M. Mills, Neil A. Fox, Heinrich J. K. Hoerber, and Charl F. J. Faul**

Imaging the Predicted Isomerism of Oligo(aniline)s: a Scanning Tunneling Microscopy Study

James O. Thomas, Hugo D. Andrade, Benjamin M. Mills, Neil A. Fox, Heinrich J. K. Hoerber* and Charl F. J. Faul*

Supporting Information

Contents

1 Experimental Section 1
2 Synthesis and Characterisation 2
2.1 Ph/C_{12} TANI LEB (S2) 2
2.2 Ph/C_{12} TANI EB (S3) 3
2.3 Ph/Ph TANI LEB (S4) 3
2.4 Ph/Ph TANI EB (S5) 4
3 Gas-phase DFT 4
3.1 EB Ph/Ph TANI 4
3.2 Ph/C_{12} TANI 6
4 References 7

1 Experimental Section

An Omicron VT AFM XA, operating at a base pressure of $5x10^{-11}$ mBar, was used to carry out the scanning tunnelling microscopy. Atomically flat, single crystal Cu(110) was prepared by sputter (Ar^+, 600 eV, 30 min) and anneal (900 K, 30 min) cycles. After thorough outgassing, Ph/Ph TANI and Ph/C_{12} TANI were deposited by thermal evaporation onto the substrate held at room temperature. STM tips were electrochemically etched tungsten wire that, after introduction to UHV, were sputtered (Ar^+, 600 eV, 2 min) to remove oxide and other contaminants. Experiments took place either at 300 K, or using a liquid helium flow cryostat, at 30 K.

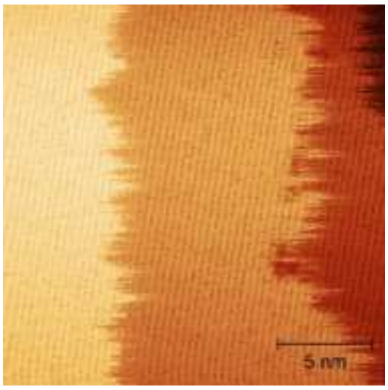

Figure S1. Cu(110) substrate imaged at 300 K, before evaporation. Imaging conditions: I = 5.2 nA, V = 0.7 V.

All DFT calculations were run using Gaussian09 software.[1] Optimized molecular structures and energies were found using the B3LYP functional and a 6-31G (d) basis set. Energies and molecular structures are presented below in Figure S2 and S3. Angles and distances were measured using GaussView software for the calculated structures, and using either Gwyddion[2] or ImageJ[3] software for the STM images.

2 Synthesis and Characterisation

2.1 Ph/C_{12} TANI LEB (S2)

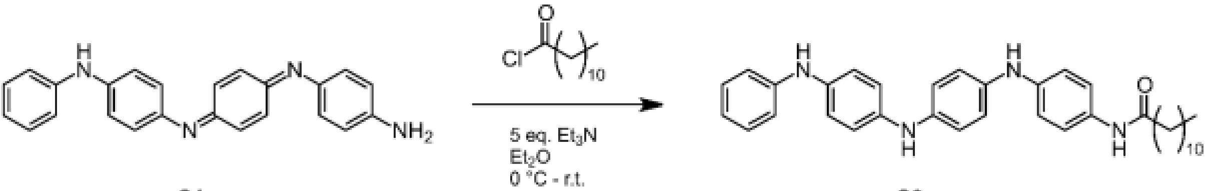

S1 **S2**

Scheme S1. Conditions for preparation of LEB Ph/C_{12} TANI from EB Ph/NH_2 TANI.

The synthesis of **S2** is based on a method reported in the literature.[4] Ph/NH_2 TANI EB, **S1**,[5] (200 mg, 0.5 mmol, 1 eq.) and triethylamine (253 mg, 347 µL, 2.5 mmol, 5 eq.) were dissolved in anhydrous diethyl ether (60 mL) in a nitrogen atmosphere and cooled to 0 °C. Lauroyl chloride (109 mg, 116 µL, 0.5 mmol, 1 eq.) in anhydrous diethyl ether (20 mL) was added drop-wise with stirring. The reaction mixture was allowed to warm to room temperature and left to react overnight. A precipitate formed and was collected by centrifugation and washed with diethyl ether until TLC analysis indicated the absence of unreacted starting material. The solid was dissolved in DMF (25 mL) and phenylhydrazine (54 mg, 0.5 mmol) was added. After 2 h, the solution was poured into deionized water (250 mL) and the precipitate was collected by centrifugation. The product **S2** was obtained in the leucoemeraldine base (LEB) state as a grey powder (211 mg, 77% yield): m.p. 202.4-205.0 °C; ^1H NMR (400 MHz, DMSO-d_6, 25 °C) δ = 9.60 ppm (s, 1H, CON*H*), 7.76 (s, 1H, N*H*), 7.67 (s, 1H, N*H*), 7.63 (s, 1H, N*H*), 7.37 (d, J = 8.9 Hz, 2H, Ar*H*), 7.14 (dd, J = 8.6, 7.3 Hz, 2H, Ar*H*), 6.99-6.86 (m, 12H, Ar*H*), 6.67 (t, 1H, J = 7.3 Hz), 2.23 (t, 2H, J = 7.3 Hz), 1.62-1.51 (m, 2H), 1.32-1.17 (m, 16H), 0.85 (t, 3H, J = 6.9 Hz) ppm; ^{13}C NMR (100 MHz, DMSO-d_6, 25 °C) 171.1, 146.0, 129.6, 121.2, 119.8, 118.8, 118.5, 118.0, 116.1, 115.1, 31.9, 29.6, 29.6, 29.5, 29.4, 29.3, 29.2, 26.5, 25.2, 22.7 ppm; IR (neat, cm^{-1}): 3377, 3369, 3325, 2918, 2850, 1656,

1602, 1530, 1516, 1497, 1302, 1221, 1172, 817, 744, 694; Anal. calcd for $C_{36}H_{44}N_4O$: C, 78.79; H, 8.08; N, 10.21. Found: C, 79.79; H, 8.18; N, 10.12. HRMS Calcd for $C_{36}H_{44}N_4O$: 548.3515. Found: 548.3510.

2.2 Ph/C_{12} TANI EB (S3)

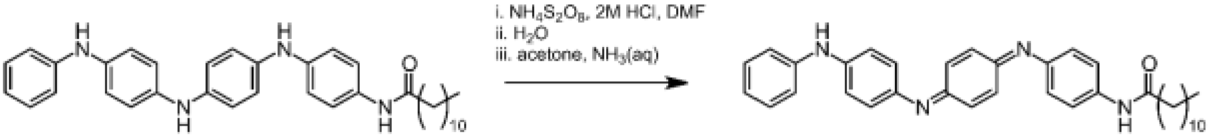

S2 **S3**

Scheme S2. Conditions for partial oxidation of LEB Ph/C_{12} TANI to EB Ph/C_{12} TANI.

S2 was oxidised to **S3** based on a previously reported method.[5] **S2** (219 mg, 0.4 mmol, 1 eq.) was dissolved in DMF (20 mL) and ammonium persulfate (91 mg, 0.4 mmol, 1 eq.) in hydrochloric acid (2M, 20 mL) was added dropwise. After stirring for 30 min, the solution was poured into deionised water (100 mL) and a dark green precipitate formed. The precipitate was isolated by centrifugation and deprotonated to the EB state with a mixture of aqueous ammonia (2M, 30 mL) and acetone (200 mL) and stirred for a further 6 h. The acetone was evaporated and the residue was filtered to afford the product **S3** as a purple powder (216 mg, 99% yield): Anal. calcd. for $C_{36}H_{42}N_4O$: C, 79.08; H, 7.74; N, 10.25. Found: C, 80.57; H, 8.45; N, 10.89. HRMS calcd. for $C_{36}H_{42}N_4O$: 546.3359. Found 546.3353.

2.3 Ph/Ph TANI LEB (S4)

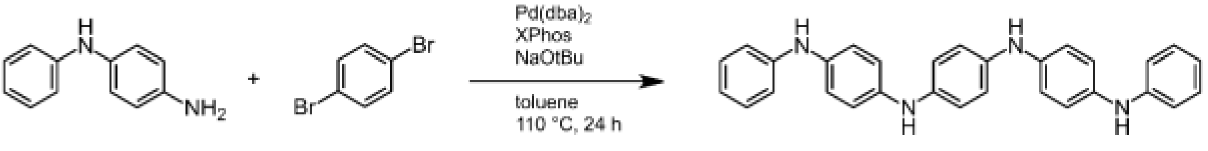

S4

Scheme S3. Preparation method for LEB Ph/Ph TANI using Buchwald-Hartwig cross coupling.

S4 was synthesised based on a previously reported method.[5] *N*-Phenyl-1,4-phenylenediamine (242 mg, 1.31 mmol, 2.1 eq), Pd(dba)$_2$ (21.6 mg, 0.04 mmol, 6 mol%), XPhos (26.2 mg, 0.06 mmol, 9 mol%), 1,4-dibromobenzene (148 mg, 0.63 mmol, 1 eq.) and sodium *tert*-butoxide (180 mg, 1.88 mmol, 3 eq.) were combined under nitrogen, dissolved in anhydrous toluene (20 mL) and refluxed at 110 °C with stirring. After 24 h, TLC analysis indicated the complete consumption of the dibromide starting material. The reaction mixture was allowed to cool to room temperature, the product was isolated by Büchner filtration and washed with cold toluene (3 × 5 mL). The resulting solid was dried under vacuum for 24 h to afford the product **S4** in the reduced LEB state as a grey powder (228 mg, 0.52 mmol, 82% yield). Analytical data were in accordance with literature values:[5] m.p. 250.1-250.9 °C; ^{1}H NMR (400 MHz, DMSO-d_6, 25 °C) δ = 7.75 (s, 2H, N*H*), 7.60 (s, 2H, N*H*), 7.13 (m, 4H, Ar*H*), 6.97 (m, 4H, Ar*H*), 6.95-6.87 (m, 12H, Ar*H*), 6.67 (tt, 2H, Ar*H*) ppm; ^{13}C NMR (100 MHz, DMSO-d_6, 25 °C) 145.5, 139.0, 137.0, 134.7, 129.1, 120.8, 118.6, 117.9, 117.2, 114.5. Anal. calcd for $C_{30}H_{26}N_4$: C, 81.42; H, 5.92; N, 12.66. Found: C, 81.60; H, 5.99; N, 12.41. HRMS Calcd for $C_{30}H_{26}N_4$: 442.2157. Found: 442.2161.

2.4 Ph/Ph TANI EB (S5)

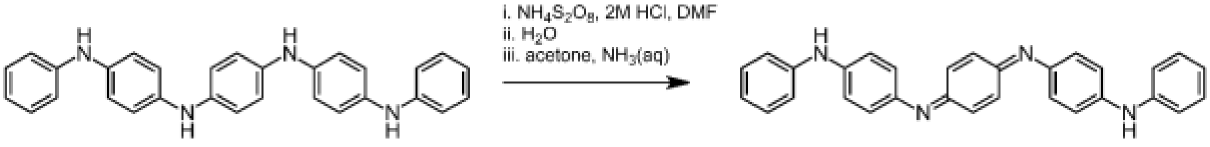

S4 **S5**

Scheme S4. Partial oxidation of LEB Ph/Ph TANI to EB Ph/Ph TANI.

S4 was oxidised to **S5** based a previously reported method.[5] **S4** (221 mg, 0.5 mmol, 1 eq.) was dissolved in DMF (50 mL) and a solution of ammonium persulfate (114 mg, 0.5 mmol, 1 eq.) in hydrochloric acid (2M, 20 mL) was added dropwise and stirred for 30 min. The solution was poured into deionised water (250 mL) and stirred. After 15 min, the precipitate was isolated by centrifugation, dedoped with a mixture of acetone (200 mL) and aqueous ammonia (2M, 50 mL) and stirred for a further 30 min. The acetone was evaporated and the remaining aqueous suspension was filtered and dried overnight in a vacuum oven at room temperature to give the product **S5** in the half-oxidised (emeraldine base) state as a purple powder (198 mg, 90% yield). HRMS Calcd for $[M+H]^+$, $C_{30}H_{24}N_4$: 441.2074. Found: 441.2073.

3 Gas-phase DFT

3.1 EB Ph/Ph TANI

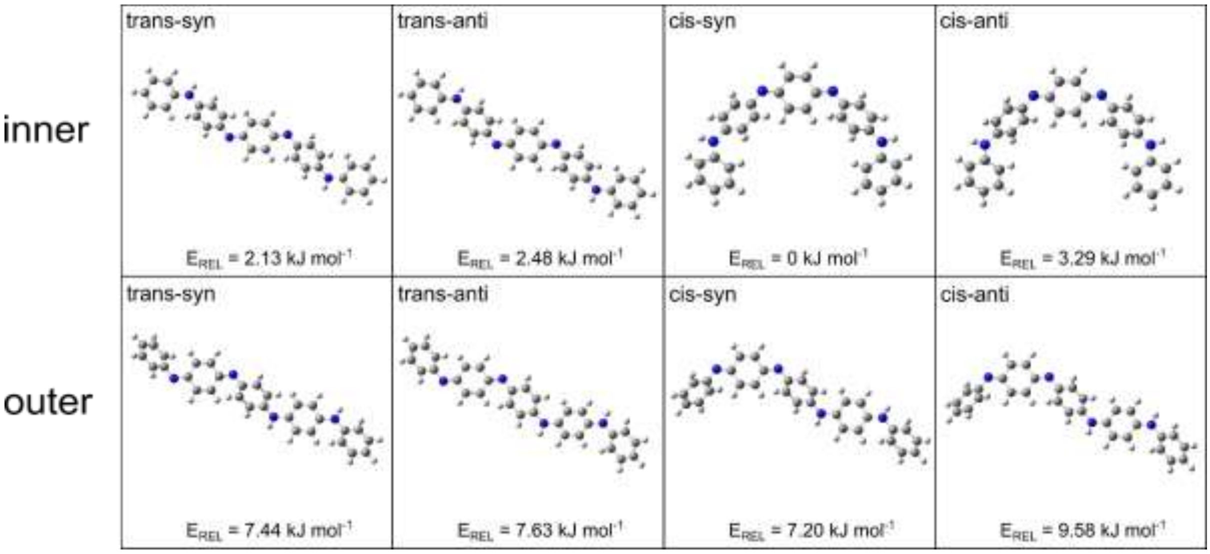

Figure S2. DFT-optimised structures of eight isomers of EB Ph/Ph TANI, including positional, *cis*/*trans* and conformational isomerism. Energies are given relative to the most stable, inner-*cis-syn* isomer.

The purpose of the DFT calculations is to visualise optimised molecular structures and therefore aid in the assignment of the molecular shapes presented in the STM images as *cis* and *trans* isomers of TANIs. Structures and energies of eight possible isomers of Ph/Ph TANI are shown in Figure S2; energies are presented relative to the most stable *cis*/*syn* inner isomer. A detailed discussion and calculations of transition states and energy barriers are beyond the scope of this study. However, barriers to positional isomerism involve a chemical change

along the backbone and therefore would be large. NMR spectra of EB Ph/Ph TANI at room temperature evolve with time, suggesting a slow, as yet, unidentified isomerism process.[6] The barrier to *cis*/*trans* rotation of the smaller model compound phenyl-capped dianiline has been estimated at 88 kJ mol^{-1}, although it was concluded by the authors of that study that there were multiple processes involved in the isomerism, and that it was catalysed by acid impurities in the solvent.

In addition to positional and *cis*/*trans* isomerism, conformational isomerism with respect to benzenoid rings either side of the quinoid ring leads to *syn/anti* isomers. The optimised structures show the flanking benzenoid rings adopt angles of ± 40° with respect to the quinoid ring. Rotation can also occur around the secondary amine groups, leading to additional local energy minima and is likely to contribute to the difficulty of imaging these isomers at room temperature.

3.2 Ph/C_{12} TANI

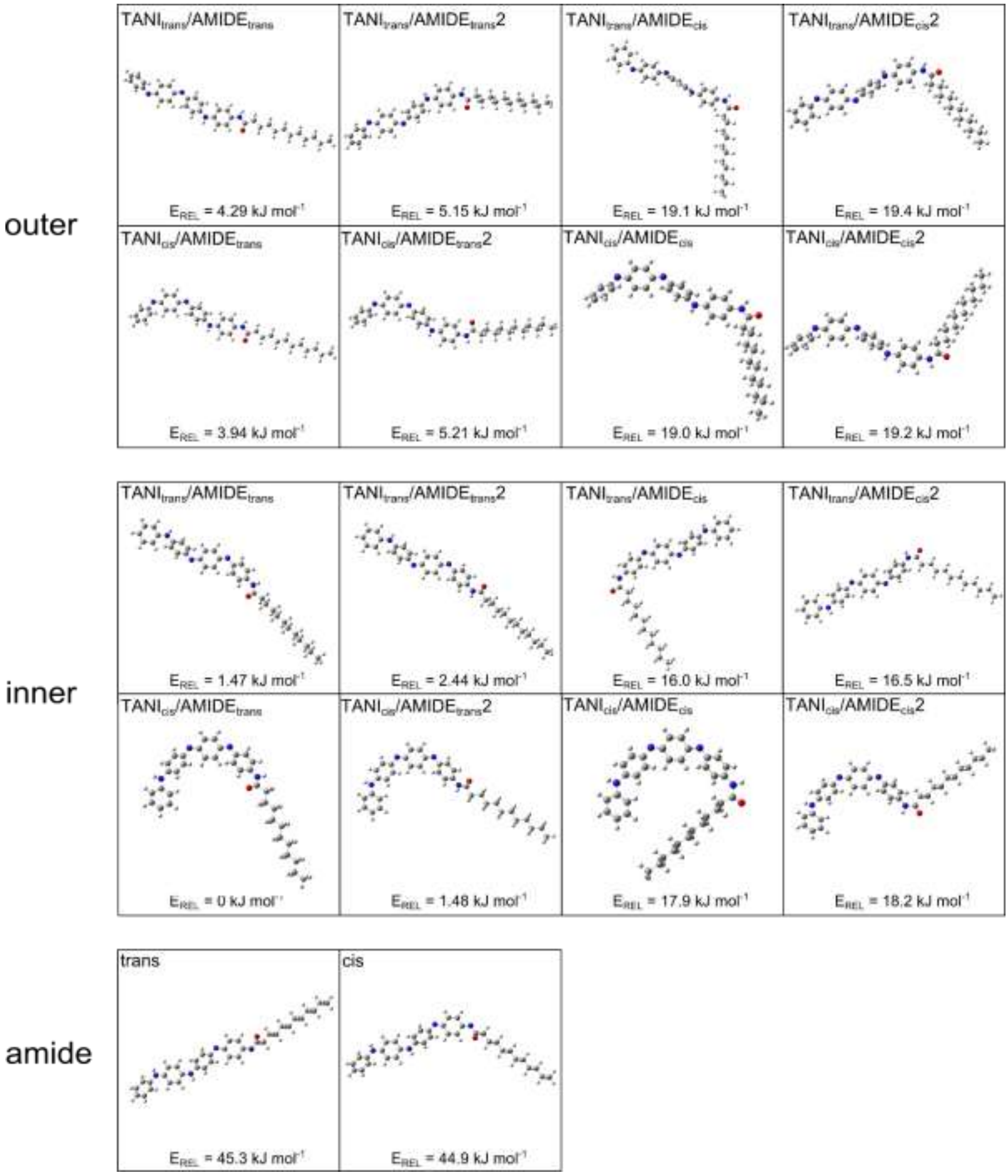

Figure S3. DFT-optimised structures and energies for a number of EB Ph/C_{12} TANI isomers. Isomers are defined by the location of the quinoid ring (inner/outer/amide), then by the *cis*/*trans* isomerism possible at both the quinoid ring (TANI$_{cis/trans}$) and the amide linker (AMIDE$_{cis/trans}$). Two orientations of the alkyl chain are shown due to rotation of the amide bond.

Ph/C_{12} has additional isomers compared with Ph/Ph TANI. Firstly three positions are now possible for the position of the quinoid ring, displayed in Figure S3: inner, outer and amide. However, due the double bond character already present in the amide linker in the LEB state Ph/C_{12} TANI and the large energy increases of ~ 45 kJ mol^{-1}, it seems likely that the amide positional isomer is present in very minor concentrations. For the inner and outer positional isomers, not only is *cis*/*trans* isomerism possible around the quinoid ring, but also around the amide linker. Whilst in nature amides exist almost exclusively in the *trans* configuration, due to the

non-equilibrium conditions of sample preparation, it is shown in Figure 4 that both $AMIDE_{cis}$ and $AMIDE_{trans}$ isomers exist, despite being typically, 15-18 kJ mol^{-1} higher in energy. With this in mind, Figure S3 displays a non-exhaustive list of the possible isomers, limited to inner/outer positional isomerism of the quinoid ring, *cis*/*trans* with respect to the quinoid ring (TANI), *cis*/*trans* with respect to the amide linker (AMIDE). Each of these eight (inner/outer) $TANI_{(cis/trans)}AMIDE_{(cis/trans)}$ isomers is displayed with two possible orientations of the amide bond, and is displayed as the *syn* isomer with respect to the angles of the benzenoid rings surrounding the quinoid ring. These isomers are chosen in order to display the large variety of molecular shapes possible for Ph/C_{12} TANI. The alkyl chain has been modelled as straight for all isomers, however when physisorbed to the Cu(110) surface this will not necessarily be the case.

4 References

[1] M. J. Frisch, G. W. Trucks, H. B. Schlegel, G. E. Scuseria, M. A. Robb, J. R. Cheeseman, G. Scalmani, V. Barone, B. Mennucci, G. A. Petersson, H. Nakatsuji, M. Caricato, X. Li, H. P. Hratchian, A. F. Izmaylov, J. Bloino, G. Zheng, J. L. Sonnenberg, M. Hada, M. Ehara, K. Toyota, R. Fukuda, J. Hasegawa, M. Ishida, T. Nakajima, Y. Honda, O. Kitao, H. Nakai, T. Vreven, J. A. Montgomery Jr., J. E. Peralta, F. Ogliaro, M. J. Bearpark, J. Heyd, E. N. Brothers, K. N. Kudin, V. N. Staroverov, R. Kobayashi, J. Normand, K. Raghavachari, A. P. Rendell, J. C. Burant, S. S. Iyengar, J. Tomasi, M. Cossi, N. Rega, N. J. Millam, M. Klene, J. E. Knox, J. B. Cross, V. Bakken, C. Adamo, J. Jaramillo, R. Gomperts, R. E. Stratmann, O. Yazyev, A. J. Austin, R. Cammi, C. Pomelli, J. W. Ochterski, R. L. Martin, K. Morokuma, V. G. Zakrzewski, G. A. Voth, P. Salvador, J. J. Dannenberg, S. Dapprich, A. D. Daniels, Ö. Farkas, J. B. Foresman, J. V. Ortiz, J. Cioslowski, D. J. Fox, Gaussian, Inc., Wallingford, CT, USA, **2009**.

[2] D. Nečas, P. Klapetek, *Centr. Eur. J. Phys.* **2012**, *10*, 181-188.

[3] C. A. Schneider, W. S. Rasband, K. W. Eliceiri, *Nat. Meth.* **2012**, *9*, 671-675.

[4] Z. Shao, Z. Yu, J. Hu, S. Chandrasekaran, D. M. Lindsay, Z. Wei, C. F. J. Faul, *J. Mater. Chem.* **2012**, *22*, 16230-16234.

[5] Z. Shao, P. Rannou, S. Sadki, N. Fey, D. M. Lindsay, C. F. J. Faul, *Chem. Eur. J.* **2011**, *17*, 12512-12521.

[6] A. G. MacDiarmid, Y. Zhou, J. Feng, *Synth. Met.* **1999**, *100*, 131-140.
